# Uncovering the roles of the scaffolding protein CsoS2 in mediating the assembly and shape of the α-carboxysome shell

**DOI:** 10.1128/mbio.01358-24

**Published:** 2024-08-29

**Authors:** Tianpei Li, Taiyu Chen, Ping Chang, Xingwu Ge, Vincent Chriscoli, Gregory F. Dykes, Qiang Wang, Lu-Ning Liu

**Affiliations:** 1 Institute of Systems, Molecular and Integrative Biology, University of Liverpool, Liverpool, United Kingdom; 2 State Key Laboratory of Crop Stress Adaptation and Improvement, School of Life Sciences, Henan University, Kaifeng, China; 3 MOE Key Laboratory of Evolution and Marine Biodiversity, Frontiers Science Center for Deep Ocean Multispheres and Earth System & College of Marine Life Sciences, Ocean University of China, Qingdao, China; Korea Advanced Institute of Science and Technology, Daejeon, South Korea; University of Southern Mississippi, Hattiesburg, Mississippi, USA

**Keywords:** bacterial microcompartment, carboxysome, self-assembly, encapsulation, structurally disordered protein

## Abstract

Carboxysomes are proteinaceous organelles featuring icosahedral protein shells that enclose the carbon-fixing enzymes, ribulose-1,5-bisphosphate carboxylase oxygenase (Rubisco), along with carbonic anhydrase. The intrinsically disordered scaffolding protein CsoS2 plays a vital role in the construction of α-carboxysomes through bridging the shell and cargo enzymes. The N-terminal domain of CsoS2 binds Rubisco and facilitates Rubisco packaging within the α-carboxysome, whereas the C-terminal domain of CsoS2 (CsoS2-C) anchors to the shell and promotes shell assembly. However, the role of the middle region of CsoS2 (CsoS2-M) has remained elusive. Here, we conducted in-depth examinations on the function of CsoS2-M in the assembly of the α-carboxysome shell by generating a series of recombinant shell variants in the absence of cargos. Our results reveal that CsoS2-M assists CsoS2-C in the assembly of the α-carboxysome shell and plays an important role in shaping the α-carboxysome shell through enhancing the association of shell proteins on both the facet-facet interfaces and flat shell facets. Moreover, CsoS2-M is responsible for recruiting the C-terminal truncated isoform of CsoS2, CsoS2A, into α-carboxysomes, which is crucial for Rubisco encapsulation and packaging. This study not only deepens our knowledge of how the carboxysome shell is constructed and regulated but also lays the groundwork for engineering and repurposing carboxysome-based nanostructures for diverse biotechnological purposes.

**IMPORTANCE:**

Carboxysomes are a paradigm of organelle-like structures in cyanobacteria and many proteobacteria. These nanoscale compartments enclose Rubisco and carbonic anhydrase within an icosahedral virus-like shell to improve CO_2_ fixation, playing a vital role in the global carbon cycle. Understanding how the carboxysomes are formed is not only important for basic research studies but also holds promise for repurposing carboxysomes in bioengineering applications. In this study, we focuses on a specific scaffolding protein called CsoS2, which is involved in facilitating the assembly of α-type carboxysomes. By deciphering the functions of different parts of CsoS2, especially its middle region, we provide new insights into how CsoS2 drives the stepwise assembly of the carboxysome at the molecular level. This knowledge will guide the rational design and reprogramming of carboxysome nanostructures for many biotechnological applications.

## INTRODUCTION

Carboxysomes (CBs) are specialized organelle-like proteinaceous microcompartments ubiquitous in cyanobacteria and some proteobacteria, playing a pivotal role in CO_2_ fixation (1, 2). Diverging from eukaryotic organelles, CBs are entirely proteinaceous, featuring a polyhedral shell and cargo enzymes crucial for CO_2_ fixation (3). The proteinaceous shell comprises primarily three groups of building blocks, including hexamers and trimers that form the facets (4–6), and pentamers that cap the vertices (7, 8). In addition, the scaffolding proteins bridge the shell and cargo enzymes, mediating the assembly of CBs (9–12). Through self-assembly *in vivo*, thousands of these building blocks form a highly ordered icosahedral shell, sequestering ribulose-1,5-bisphosphate carboxylase oxygenase (Rubisco) and carbonic anhydrase for the construction of a functional entity (1). Moreover, the porous shell functions as a barrier, selectively modulating the influx and efflux of metabolites, significantly facilitating the catalytic performance of the interior enzymes (4, 13–17). The self-assembly, permeability, and catalytic enhancement properties of CBs make them an appealing bioengineering target for applications in crop engineering, biofuel production, metabolic enhancement, and therapeutics (18–21).

CBs are classified into two categories: α-CBs that are primarily encoded by the *cso* operon and β-CBs that are primarily encoded by the *ccm* operon (22, 23). Unlike β-CBs that undertake a “Cargo first” assembly pathway (24, 25), the self-assembly of α-CBs is presumed to follow a “Shell first” or “Concomitant shell−core assembly” mode (26–28). This unique assembly pathway offers α-CBs with greater potential to engineer empty shell structures that can be used to enclose foreign cargos and molecules for generation of new nanobioreactors and scaffolding nanomaterials. Previous studies have demonstrated the possibilities of engineering intact α-CBs (29–31), entire or simplified α-CB shells (20, 32–35), and α-CB-based nanobioreactors in *Escherichia coli* (20, 21), as well as transforming α-CBs into plant chloroplasts for boosting carbon fixation (19, 36). In this context, understanding the exact structural organization and assembly mechanisms of α-CBs and α-CB shells is fundamental for rational design and reprogramming of CB-inspired nanostructures.

**Fig 1:**
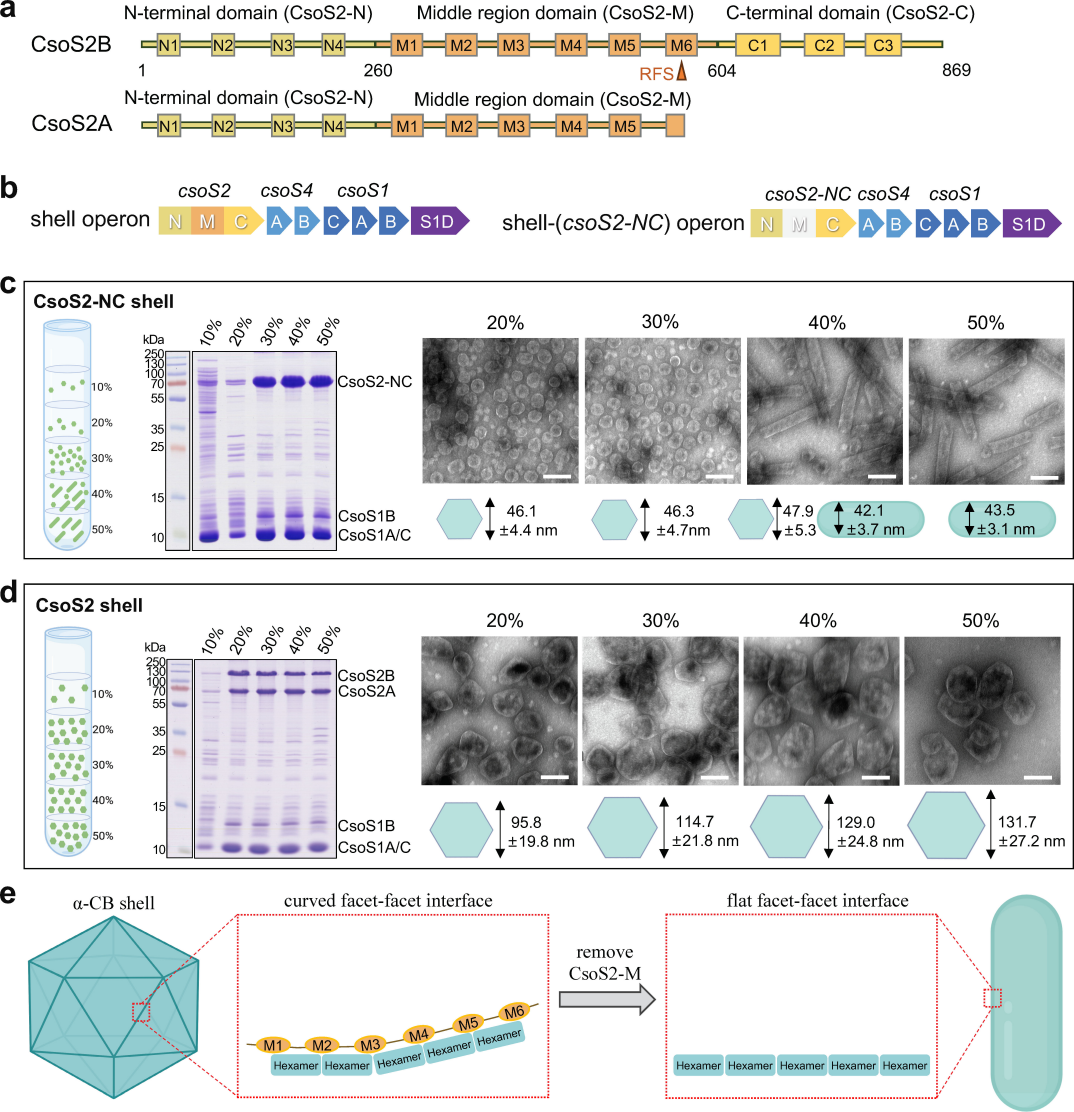
CsoS2-M defines the size and shape of the α-CB shells. (**a**) The domain arrangement of CsoS2. The N-terminal (CsoS2-N), middle (CsoS2-M), and C-terminal (CsoS2-C) domains are colored sand, orange, and yellow, respectively. A ribosomal frameshift site (RFS) located at the sixth repeat (**M6**) of CsoS2-M leads to the production of a short CsoS2A isoform. (**b**) The genetic arrangements of shell and shell-(*csoS2-NC*) operons, with the deleted region colored gray. (**c–d**) Cartoon models showing the distribution of CsoS2-NC shell and tubular assemblies (**c**), and CsoS2 shells (with unmodified CsoS2) (**d**) across 10%–50% sucrose fractions after ultracentrifugation (left panel). SDS-PAGE of CsoS2-NC shell and tubular assemblies (**c**) and CsoS2 shells (**d**) isolated from 10 to 50% sucrose fractions (middle panel). Transmission EM images displaying CsoS2-NC shell and tubular assemblies (**c**) and CsoS2 shells (**d**) in 20%–50% sucrose fractions, respectively (right panel). The average diameters of shells and widths of tubular structures in various sucrose fractions are depicted below the EM images. Scale bar: 100 nm. (**e**) A cartoon model illustrating a possible mechanism by which CsoS2-M defines the shell curvature. CsoS2-M is likely involved in adjusting the tilt angles between neighboring shell facets by enhancing the association of shell proteins on the facet-facet interfaces.

Recent studies using x-ray crystallography and cryo-electron microscopy (cryo-EM) have provided valuable insights into the building principles of α-CBs, in particular the crucial role of the scaffolding protein CsoS2 in orchestrating the assembly of shell proteins and cargos (9, 33, 37–40). CsoS2 comprises three distinct regions: an N-terminal region (CsoS2-N), a middle region (CsoS2-M), and a C-terminal region (CsoS2-C) (Fig. 1a). Moreover, each region contains various repetitive fragments. Specifically, in the model chemoautotrophic bacterium *Halothiobacillus neapolitanus*, CsoS2-N includes four repeats, CsoS2-M contains six repeats, and CsoS2-C encompasses three repeats. It has been demonstrated that CsoS2-N binds with Rubisco through multivalent interactions, playing a role in recruiting Rubisco (9, 38), whereas CsoS2-C binds with shell proteins via its C-repeats, functioning as a “molecular thread” to stitch multiple shell proteins, thereby facilitating shell assembly (32). CsoS2-M has been suggested to be important in determining the size of α-CBs (41). In addition, a recent study on the α-CB from the marine α-cyanobacterium *Prochlorococcus* MED4 showed that CsoS2-M binds to multiple hexamers on the shell facets and facet-facet junctions through multivalent interactions in, implying an important role of CsoS2-M in shaping the structure of α-CBs (40).

CsoS2 in many microorganisms, including *H. neapolitanus*, contains a ribosomal frameshift site (RFS) within CsoS2-M, leading to the premature termination and production of a shorter isoform CsoS2A, in addition to a full-length isoform CsoS2B (42) (Fig. 1a). Compared to CsoS2B, CsoS2A lacks CsoS2-C that is crucial for binding with the α-CB shell. How CsoS2A is incorporated within the α-CB, how it coordinates with CsoS2B, as well as the precise roles of CsoS2-M and CsoS2A in determining shell formation and architecture remain elusive.

Here, by generating a series of recombinant α-CBs shells derived from *H. neapolitanus*, we systematically evaluated the roles of individual domains of CsoS2, without cargo proteins, in modulating the shell formation. We show that CsoS2-M assists CsoS2-C in enhancing the connections between hexamers that are distal from the shell vertices, thereby playing a dominant role in determining the size and shape of α-CB shells. These findings enable us to develop a model to elucidate the mechanisms by which CsoS2 facilitates the assembly of the α-CB shell. This study advances our knowledge of the self-assembly and structural basis of α-CBs, which lays the groundwork for future engineering and refinement of α-CBs for various biotechnological and biomedical applications.

## RESULTS

### CsoS2-M plays a role in determining the shell size and morphology

In previous work, we have constructed recombinant α-CB shells with an average size of ~120 nm in *E. coli*, by expressing a shell operon derived from *H. neapolitanus* (20). This shell operon consists of genes encoding CsoS2, pentamers CsoS4A/4B, hexamers CsoS1A/1B/1C, and trimers CsoS1D (Fig. 1b). To investigate the role of CsoS2-M during shell assembly, we generated a shell-(*csoS2-NC*) operon by deleting only the nucleotide sequences encoding CsoS2-M from the shell operon (Fig. 1b). The shell-(*csoS2-NC*) operon was expressed in *E. coli*, and the resulting shell assemblies were purified by sucrose gradient centrifugation. SDS-polyacrylamide gel electrophoresis (SDS-PAGE) confirmed the presence of CsoS2-NC and CsoS1A/B/C in various sucrose fractions, with a predominant distribution in the 30%–50% sucrose fractions (Fig. 1c). Surprisingly, despite the same protein composition in the 20%–50% sucrose fractions identified by SDS-PAGE, EM revealed distinct tubular structures with a mean width of 42.1 ± 3.7 or 43.5 ± 3.1 nm in 40% and 50% sucrose fractions, whereas polyhedral shells (46.8 ± 4.9 nm on average in diameter) were enriched in 20% and 30% sucrose fractions (Fig. 1c). By contrast, expression of the shell operon in *E. coli* exclusively produced polyhedral shells, despite that the shell size varies among different sucrose fractions (Fig. 1d). Moreover, the polyhedral shells (~47 nm) produced by the shell-(*csoS2-NC*) operon were remarkably smaller than the intact α-CB shells (~120 nm) (Fig. 1c and d). These results indicate that CsoS2-M plays an important role in defining the size and shape of α-CB shells.

We speculate that CsoS2-M is involved in adjusting the tilt angles between neighboring facets of the icosahedral shell (Fig. 1e). Without CsoS2-M, the interaction between hexamers situated at the curved facet-facet interface is diminished, resulting in the formation of a flat facet-facet interface and eventually the generation of low-curvature tubular structures (Fig. 1e). Consistent with this model, the cryo-EM structure of the simple and small α-CB from *Prochlorococcus* revealed that CsoS2-M binds to multiple hexamers distributed on the adjacent facets of the icosahedral shell through multivalent interactions (40).

### Roles of CsoS2-N and CsoS2-C in the shell assembly

**Fig 2:**
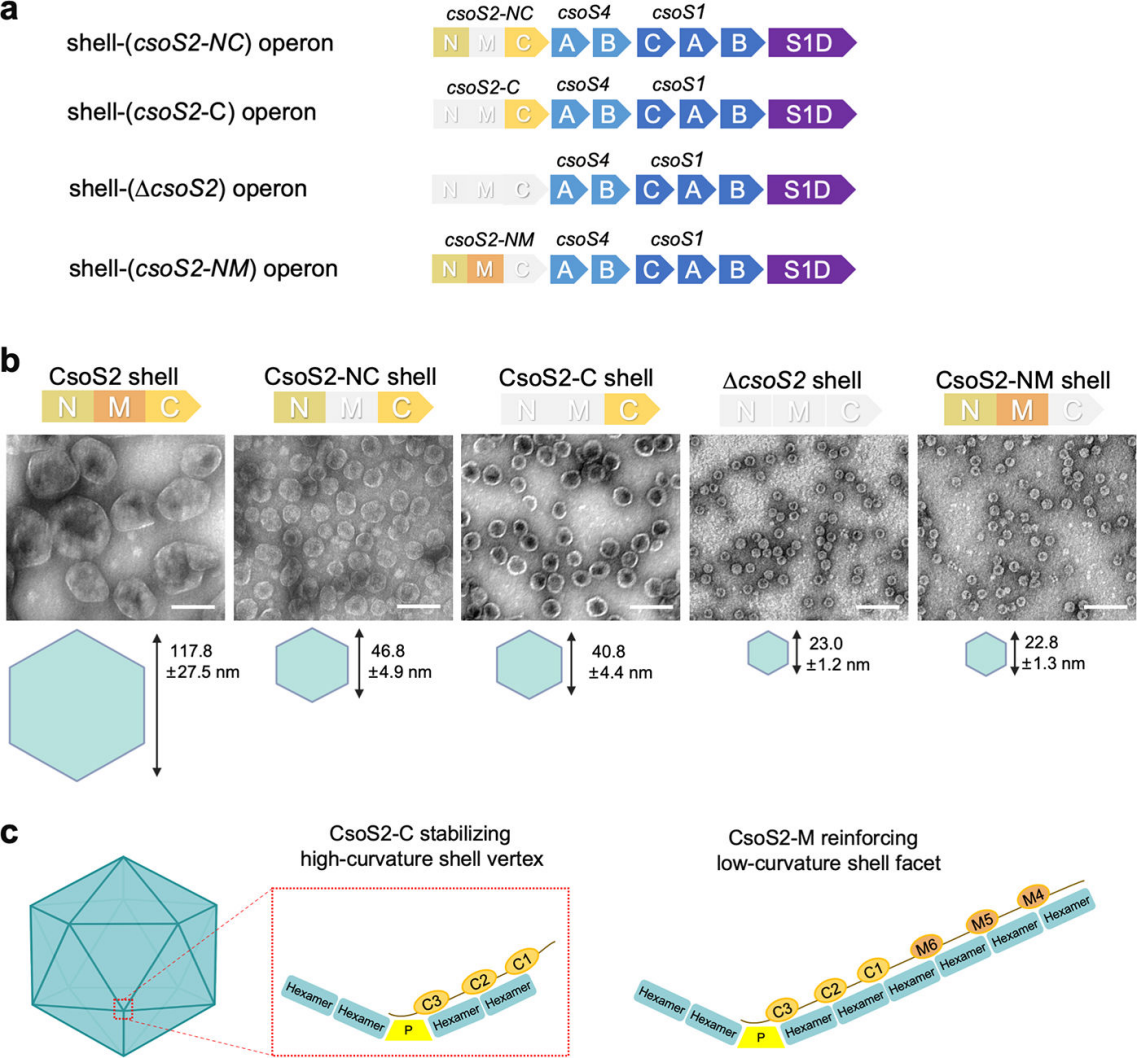
The role of individual domains of CsoS2 in modulating shell size. (**a**) The genetic arrangements of shell-(*csoS2-NC*), shell-(*csoS2-C*), shell-(∆*csoS2*), and shell-(*csoS2-NM*) operons. (**b**) Transmission EM images of CsoS2 shells, CsoS2-NC shells, CsoS2-C shells, ∆*csoS2* shells, and CsoS2-NM shells, respectively. The average sizes are displayed below the EM image. Scale bar: 100 nm. (c) A cartoon model depicting interactions of CsoS2-M and CsoS2-C with shell proteins during shell assembly; CsoS2-M assists CsoS2-C in enhancing the connections between the hexamers that are distal from the shell vertices. P, pentamer.

We also investigated the role of CsoS2-N and CsoS2-C by deleting *csoS2-N* and *csoS2-C* from the shell-(*csoS2-NC*) operon (Fig. 2a). Removing CsoS2-N alone [shell-(*csoS2-C*) operon] or both CsoS2-N and CsoS2-C [shell-(∆*csoS2*) operon] resulted in the production of polyhedral shells exclusively (Fig. 2b). The CsoS2-C shells were 40.8 ± 4.4 nm in diameter, slightly smaller than the CsoS2-NC shells (46.8 ± 4.9 nm), which implies that CsoS2-N has no substantial effect on the shell size. This supports the finding that CsoS2-N interacts with Rubisco to facilitate Rubisco encapsulation into the α-CB (9). By contrast, the ∆*csoS2* shells (23.0 ± 1.2 nm) were only half the size of the CsoS2-C shells (40.8 ± 4.4 nm) (Fig. 2b; Figure S1a and b), suggesting that CsoS2-C has a significant impact on the shell size through interactions with shell proteins. In line with this, our recent work has shown that the binding of CsoS2-C with shell proteins could result in an increase in the size of “mini-shells,” which are made up of CsoS1A hexamers and CsoS4A pentamers, from 25 nm to 37 nm (32).

To further evaluate the role of CsoS2-C in modulating the shell size, we also generated a shell-(*csoS2-NM*) operon by deleting *csoS2-C* from the shell operon (Fig. 2a). Interestingly, expression of the shell-(*csoS2-NM*) operon in *E. coli* yielded exclusively mini-shells with the diameter of 22.8 ± 1.3 nm, and CsoS2-NM was not incorporated in the purified CsoS2-NM shells (Fig. 2b; Fig. S1c). This result confirms the importance of CsoS2-C in forming shells with the diameter larger than ~23 nm and suggests that CsoS2-M alone is insufficient to drive the assembly of intact α-CB shells (~120 nm).

Given that CsoS2-C binds to the high-curvature pentamer-hexamer and hexamer-hexamer interfaces (32), it is likely that the multivalent interactions between CsoS2-C and shell proteins close to shell vertices are essential for the formation of α-CB shells and may potentially initiate the assembly of intact α-CB shells (~120 nm). CsoS2-C is probably recruited onto the shell prior to CsoS2-M, occupying the highly curved vertices where shell hexamers and pentamers meet; subsequently, CsoS2-M attachment to the shell triggers the binding of additional hexamers and creates more hexamer-hexamer interfaces that are distant from the shell vertices, resulting in the expansion of shell facets to generate intact α-CB shells (Fig. 2c).

### The repeating motifs of CsoS2-M define the shell size

**Fig 3:**
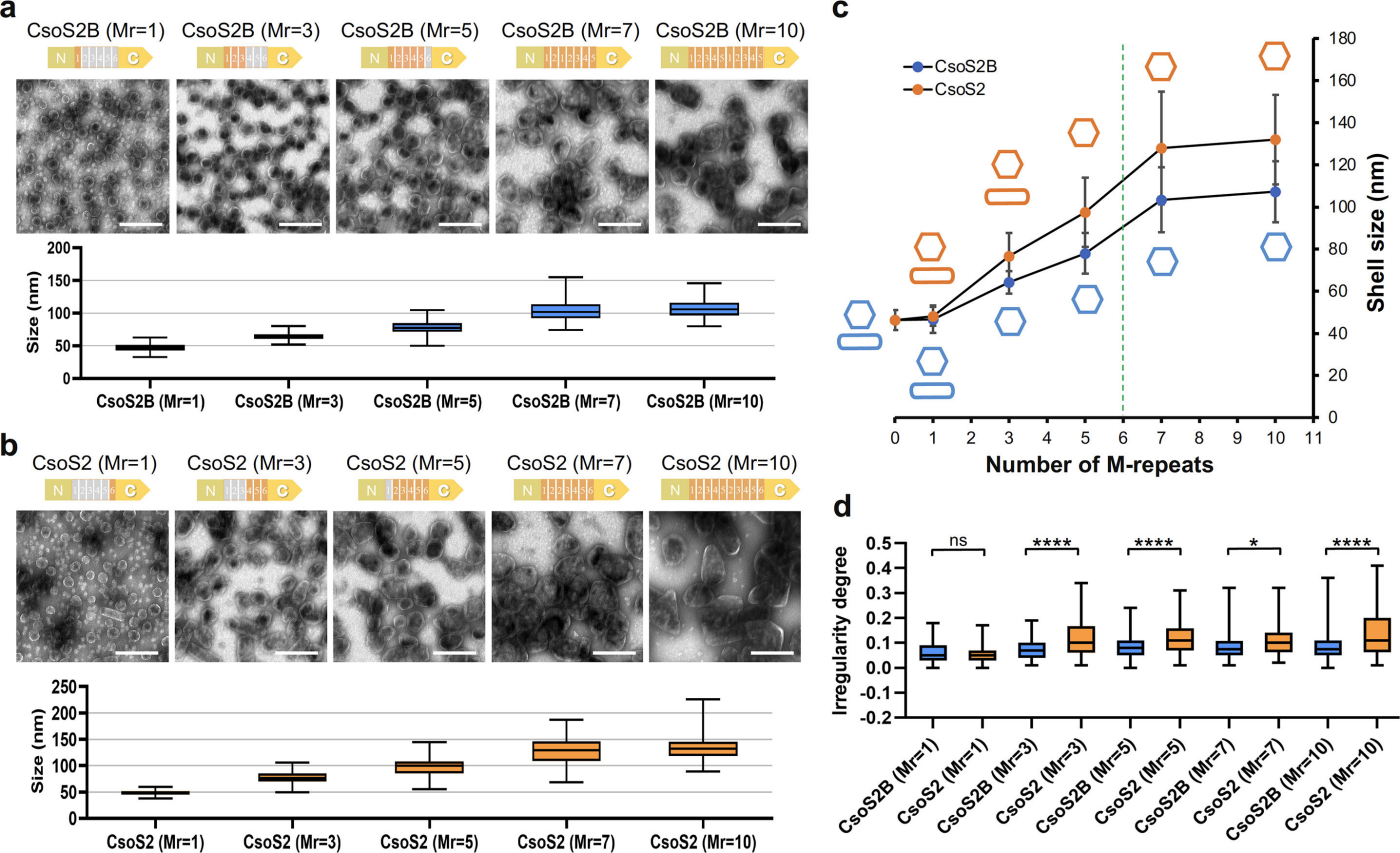
The number of M-repeats in CsoS2-M determines the shell size and shape. (**a**) A series of CsoS2B shell variants with M-repeats range from 1 (Mr = 1) to 10 (Mr = 10) in CsoS2B. Transmission EM images of each shell variant are displayed above the size distribution plot. Scale bar: 200 nm. Samples were collected from the sucrose fraction enriched with the most abundant shell proteins, as determined by SDS-PAGE (Fig. S3). (**b**) A series of CsoS2 shell variants with M-repeats range from 1 (Mr = 1) to 10 (Mr = 10) in CsoS2. Scale bar: 200 nm. (**c**) Plot of the mean diameter of various shell variants as a function of the number of M-repeats. The cartoon models colored orange or blue represent the morphology of CsoS2 or CsoS2B shell variants, respectively. Native CsoS2 contains six M-repeats, as indicated by the green dashed line, which are of biological importance for the formation of large polyhedral α-CB structures. (**d**) The irregularity degree of CsoS2B and CsoS2 shell variants. *, 0.01 ≤ *P* ≤ .05; ****, *P* ≤ 0.0001; ns, no significance (*n* = 100, two-tailed unpaired *t*-test). Box plots indicate the median (middle line in the box), 25th percentile (bottom line of the box), and 75th percentile (top line of the box).

CsoS2-M contains six repeating fragments (M1–M6), each of which is ~50 residues in length, separated by short linker sequences of 5 ~ 15 residues, and possesses three conserved [V/I/M][T/S]G motifs (Fig. 1a; Fig. S2). To delineate in-depth how CsoS2-M regulates the shell size and shape, we generated a series of CsoS2 and CsoS2B variants with varying numbers of M-repeats in the M-region (Fig. 3a and b). All the CsoS2 variants retained the RFS at the sixth M-repeat (M6), allowing the production of both CsoS2B and CsoS2A isoforms, each with a varying number of M-repeats. By contrast, the CsoS2B variants lack M6, leading to the exclusive production of the CsoS2B isoform with a varying count of M-repeats. Given the high conservation among different M-repeats, individual differences in M-repeats were not considered in the design of these CsoS2 and CsoS2B variants (Fig. S2).

The resulting shell variants were purified from *E. coli* using sucrose gradient centrifugation. SDS-PAGE verified the presence of CsoS2B variants in all the CsoS2B shell variants, and the coexistence of CsoS2B and CsoS2A isoforms in the CsoS2 shell variants, along with the main shell proteins CsoS1A/B/C (Fig. S3). An exception is the CsoS2 shell variant that contains only a single M-repeat (M6), termed CsoS2 (Mr = 1) shell, in which only CsoS2B(M6) was incorporated in the purified shell (Fig. S3b). This suggests that the short isoform CsoS2A(M6) with a single M-repeat was unable to be encapsulated within the shell. To further examine the minimal number of M-repeats required for the encapsulation of CsoS2A, we generated a construct to produce a CsoS2 (Mr = 2) shell variant that contains two M-repeats (M5–M6). SDS-PAGE showed the presence of CsoS2A(M5–M6) in the purified CsoS2 (Mr = 2) shells (Fig. S4), indicating that at least two M-repeats are required to ensure stable interactions between M-repeats and the shells for recruiting CsoS2A.

EM analysis of CsoS2B shell variants revealed that the size of the CsoS2B shell variants exhibited a significant increase, growing from 46.7 ± 6.5 nm to 107.3 ± 14.5 nm, corresponding to the rise in the number of M-repeats from 1 (Mr = 1) to 10 (Mr = 10) (Fig. 3a). Similarly, the size of the CsoS2 shell variants increased from 48.1 ± 4.5 nm to 132.0 ± 21.3 nm (Fig. 3b). Interestingly, this upward trajectory seemed to decelerate when the number of M-repeats in both CsoS2 and CsoS2B shells exceeded seven (Mr = 7) (Fig. 3c). Overall, our results indicate that the number of CsoS2-M-repeats is a key factor in determining the size of α-CB shells; larger shells are formed when the number of M-repeats increases. These findings support our hypothesis that CsoS2-M plays a role in reinforcing hexamer-hexamer interfaces that are distant from the shell vertices. More M-repeats accommodated in CsoS2-M will lead to the formation of larger shell facets and stable facet-facet interfaces, essential for the assembly of large polyhedral shells (Fig. 2c; Fig. S5). It is worth mentioning that the steady increase in the number of M-repeats did not lead to an infinite expansion of empty shells, suggesting that there should be other factors involved in defining the shell size. Consistent with our observations, an increase in the size of recombinant α-CBs that contain cargos was also found as the number of M-repeats increased (41).

We also found that the CsoS2 shell variants (containing both CsoS2A and CsoS2B) appeared to be greater in size than the CsoS2B shells with the same number of M-repeats, suggesting that CsoS2A plays a role in facilitating the assembly of larger shells (Fig. 3c). This is consistent with the previous finding that recombinant α-CBs, with cargos, containing both CsoS2 isoforms were larger than those with only CsoS2B (41). Moreover, CsoS2 (Mr = 3) featuring three M-repeats produced both polyhedral shells and tubular structures in the heavier sucrose fraction, whereas CsoS2B (Mr = 3) with three M-repeats yielded exclusively polyhedral shells (Fig. 3c; Fig. S5b). The resulting CsoS2 shells also displayed greater heterogeneity compared to the CsoS2B shells with the same number of M-repeats (Fig. 3d). These results suggest that CsoS2A is involved in governing both the shell size and curvature. The additional M-repeats of CsoS2A, which are absent in the CsoS2B shells, might coordinate with the M-repeats of CsoS2B to organizing hexamers away from the shell vertices, contributing to the further extension of the shell facet and eventually forming larger shells. This is further supported by the observations that the CsoS2 (Mr = 1) shell and CsoS2B (Mr = 1) shell, both of which have no CsoS2A, exhibit similar shell sizes (Fig. 3c; Fig. S3). On the other hand, without CsoS2-C for anchoring near the shell vertices, CsoS2A might be able to adopt more flexible binding modes with shell proteins, which leads to the higher structural heterogeneity of the CsoS2A-containing shells.

### Encapsulation of CsoS2A is independent of its interaction with CsoS2-M of CsoS2B

It was assumed that the interactions between the CsoS2-M of CsoS2A and CsoS2B ensure the recruitment of CsoS2A into the α-CB (12). To test this, we constructed a plasmid to express CsoS2A with enhanced green fluorescent protein (GFP) fused at its N-terminus (GFP-CsoS2A), and fused mCherry to the C-terminus of CsoS1B (CsoS1B-mCherry) in those shell constructs. This allowed us to visualize the distribution of CsoS2A and shell variants in *E. coli*. Additionally, to eliminate the influence of endogenous CsoS2A that may potentially compete with GFP-CsoS2A for interaction with CsoS2B, we constructed the shell-(*csoS2B-only*) operon by removing the RFS of *csoS2* (41, 42), to express CsoS2B only without CsoS2A, along with shell proteins. The formation of the CsoS2B-only shells was confirmed by SDS-PAGE and EM (Fig. S6). The CsoS2B-only shells labeled with mCherry were used for the following fluorescence co-localization analysis.

**Fig 4:**
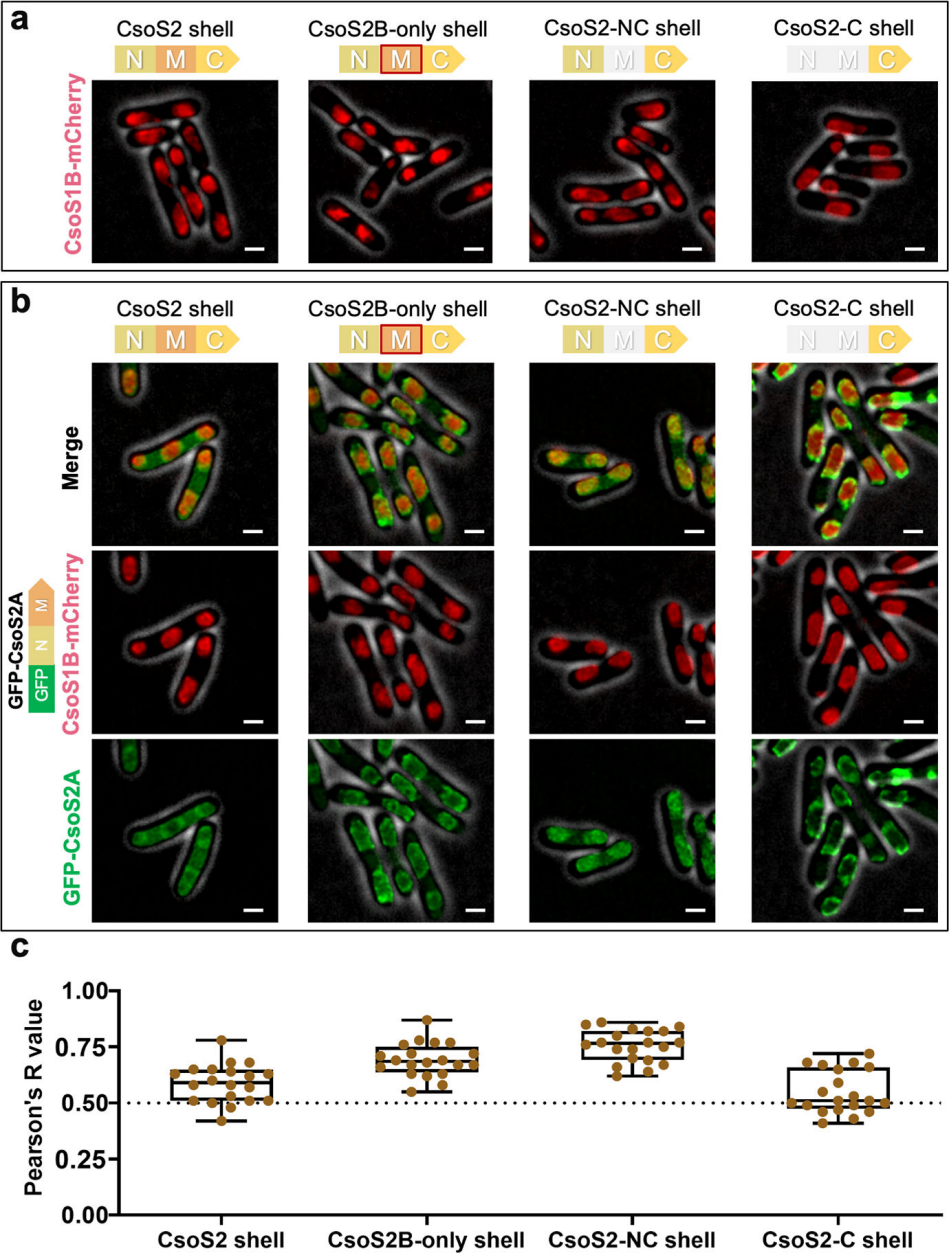
The encapsulation of CsoS2A is not mediated by interactions between CsoS2-M in CsoS2A and CsoS2B. (**a**) Confocal images of cells expressing CsoS2 shell, CsoS2B-only shell, CsoS2-NC shells, and CsoS2-C shells, respectively. CsoS1B was labeled by mCherry. Fluorescence foci (red) indicate shells. The CsoS2-M module with a red frame in the CsoS2B-only shell operon represents CsoS2-M without RFS. (**b**) Confocal images of cells co-expressing GFP-CsoS2A (green) with CsoS2 shell (red), GFP-CsoS2A with CsoS2B-only shell, GFP-CsoS2A with CsoS2-NC shell, and GFP-CsoS2A with CsoS2-C shell. Scale bar: 1 µm. (**c**) Co-localization analysis of GFP and mCherry fluorescence in (**b**) the Pearson’s R values for all the strains are 0.59 ±  0.09 (CsoS2 shell), 0.69 ±  0.07 (CsoS2B-only shell), 0.76 ±  0.07 (CsoS2-NC shell), and 0.55 ±  0.10 (CsoS2-C shell). Data are represented as mean ± SD. *n* = 20, representing the number of cells.

These mCherry-labeled shells, including the CsoS2 shells (with unmodified CsoS2), CsoS2B-only shells, CsoS2-NC shells, and CsoS2-C shells, were either expressed individually or co-expressed with GFP-CsoS2A. Confocal images of *E. coli* cells expressing different mCherry-labeled shell variants showed similar polar distribution of shell assemblies (Fig. 4a). Intriguingly, co-localization of GFP-CsoS2A (green) and CsoS1B-mCherry (red) was visualized in cells expressing the CsoS2 shells, CsoS2B-only shells, CsoS2-NC shells, or CsoS2-C shells (Fig. 4b). These data indicate that CsoS2A could be incorporated into the shells without the assistance of endogenous CsoS2-M, indicating that the encapsulation of CsoS2A was not mediated by interactions between the CsoS2-M in CsoS2B and CsoS2A. In addition, a higher degree of co-localization was found in cells expressing the CsoS2B-only shells than the CsoS2 shells (Fig. 4c). This suggests that the latter has a reduced CsoS2A-loading capacity, which might be attributed to the competition between GFP-CsoS2A and endogenous CsoS2A for the limited binding sites on the shell inner surface. The CsoS2-C shells exhibited the lowest co-localization compared to the other three types of shell variants (Fig. 4c), possibly due to the smallest size of the CsoS2-C shells, which likely results in a reduced number of docking sites for GFP-CsoS2A binding.

To further validate that the association between CsoS2-M of CsoS2A and the shell is responsible for CsoS2A encapsulation, we deleted the nucleotides encoding CsoS2-N from the plasmid expressing GFP-CsoS2A. This resulted in the generation of a GFP-fused CsoS2-M (GFP-S2M). We then co-expressed this construct with the mCherry-labeled shell variants. Confocal images revealed a comparable co-localization pattern of GFP and mCherry signals at the cell poles (Fig. S7). This suggests that CsoS2-M within CsoS2A can mediate the encapsulation of CsoS2A into the shell, likely through the binding between the conserved [V/I/M][T/S]G motifs of CsoS2-M and shell hexamers (32, 40, 43).

## DISCUSSION

**Fig 5:**
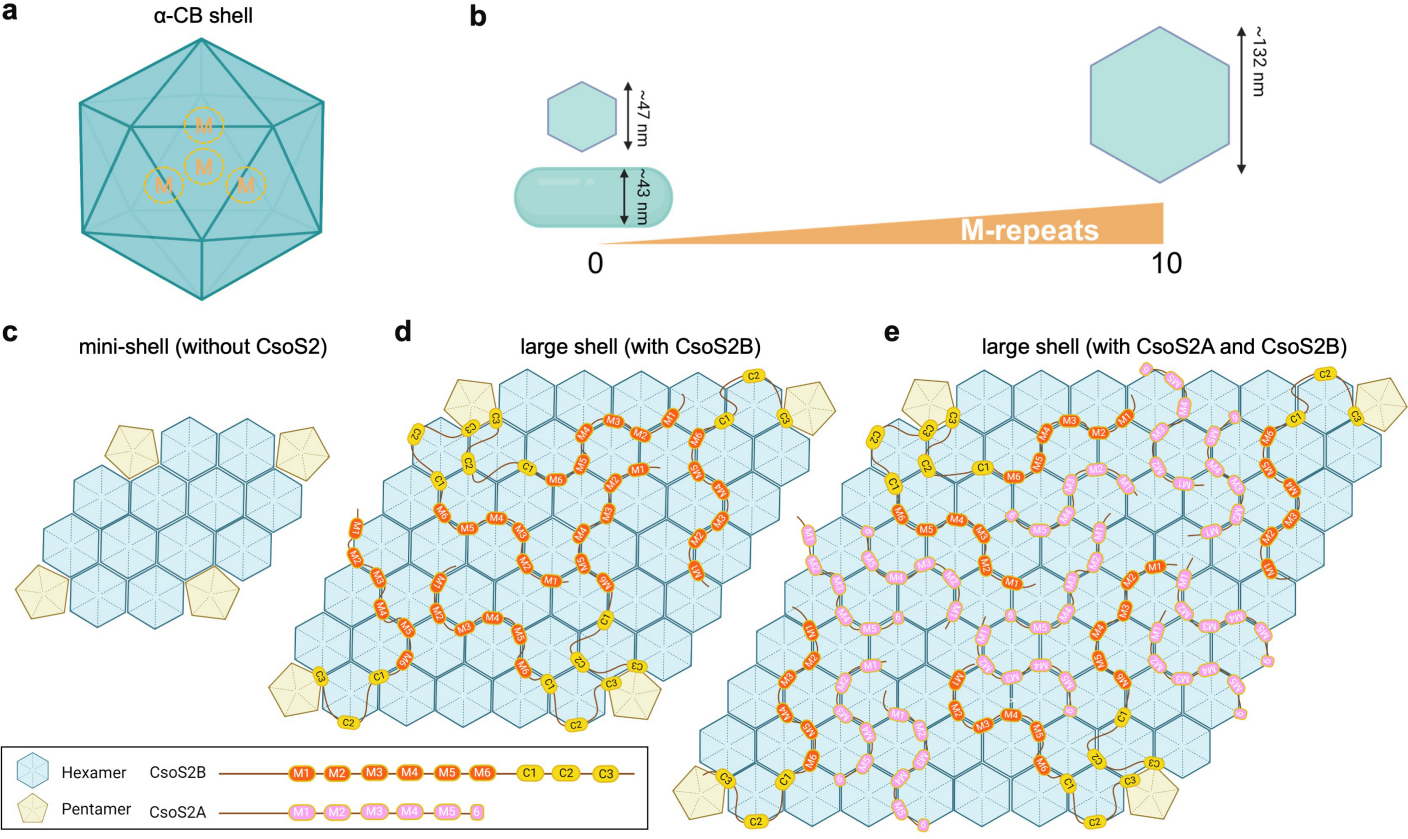
The role of CsoS2 in α-CB shell assembly. (**a**) The interaction sites of CsoS2-M on the α-CB shell. M represents CsoS2-M; the dashed circles indicate the anchoring sites of CsoS2-M on the shell. (**b**) The number of M-repeats defines the shell size and shape, with more M-repeats contributing to larger polyhedral shells. (**c**) Without CsoS2, shell proteins self-assemble into small facets, resulting in mini-shell formation. (**d**) With CsoS2B, shell proteins assemble into large facets, yielding large shells. The CsoS2-C connects the pentamer and the proximal hexamers; CsoS2-M reinforces hexamer-hexamer interfaces distal from the shell vertices. (**e**) With both CsoS2B and CsoS2A, shell proteins can form larger facets and larger shells, compared to CsoS2B-only shells. The CsoS2A adopts a flexible binding mode at the hexamer-hexamer interfaces distal from the shell vertices and, consequently, contributes to larger shell formation.

How the scaffolding protein CsoS2 governs the shell assembly and shell-cargo association is a key question not only in the fundamental studies of α-CB assembly but also in the bioengineering of CB-based nanostructures. This study enhances our understanding of the role of CsoS2, particularly its middle region CsoS2-M, in controlling the size and curvature of α-CB shells in the absence of cargos. We show that CsoS2-M plays a dominant role in shaping the α-CB shell, possibly through strengthening the hexamer-hexamer association on both the facet-facet interfaces and flat shell facets distal from the shell vertices (Fig. 5a). However, without CsoS2-C, neither CsoS2A nor CsoS2-M can independently orchestrate the assembly of the large α-CB shell (~120 nm) (Fig. 2b). More importantly, we show that the number of M-repeats in CsoS2-M plays a crucial role in determining the shell size and curvature, with a higher number of M-repeats resulting in enlarged polyhedral shells (Fig. 5b).

The recent study on *Prochlorococcus* α-CBs has revealed specific binding patterns of CsoS2 on the α-CB shell inner surface (40). However, it is important to note that the α-carboxysomes of *Halothiobacillus* and *Prochlorococcus* differ in several aspects. First, the *csoS2* gene in *Halothiobacillus* produces both CsoS2B and CsoS2A isoforms, whereas *csoS2* in *Prochlorococcus* only produces the full-length CsoS2 (44). The two forms of CsoS2 reported in *Prochlorococcus* α-CBs might be due to the variable organization of the full-length CsoS2, which merits further investigation (40). Additionally, their shell protein composition differs. *Prochlorococcus* has the simplest α-CB operon, with its α-CB containing only one type of CsoS1 hexamer, whereas *Halothiobacillus* α-CB contains three different types of hexamers, CsoS1A/B/C. These discrepancies may lead to different protein bindings and contribute to the size disparity between the two α-CBs, relatively smaller and regular α-CBs in *Prochlorococcus* (~86 nm in diameter) compared to the larger and less structurally homogeneous *Halothiobacillus* α-CBs (~120 nm in diameter).

Based on our observations, combined with recent findings revealing the interactions between CsoS2-C with shell proteins and the cryo-EM structure of a small α-CB from *Prochlorococcus* (32, 40), we propose a model to elucidate the role of CsoS2 in shaping the α-CB shell. Without CsoS2, shell proteins could only self-assemble into mini-shells with a maximal size of ~25 nm (Fig. 5c). In the presence of CsoS2B, the CsoS2-C of CsoS2B attaches to the shell hexamers that surround the pentamer, while the flexible M-repeats of CsoS2-M extend outward, acting as a hinge to link neighboring shell hexamers on both the flat facets away from shell vertices and the facet-facet interfaces. The increase in the content of CsoS2 M-repeats enables association with a higher number of shell hexamers. These structural features of the intrinsically disordered protein CsoS2 and the specific interactions of CsoS2-M with shell components facilitate the assembly of large shell facets and determine the tilt angles between adjacent shell facets, eventually promoting the formation of large polyhedral shells (Fig. 5d).

The C-terminal truncated isoform of CsoS2, CsoS2A, has been proposed to solely coordinate Rubisco packaging within the α-CB, without attaching to the shell (12). Our results show that CsoS2A can be recruited into the empty shell and its encapsulation is driven by the interactions between the CsoS2-M of CsoS2A and the shell, instead of the interactions between CsoS2A and CsoS2B. Moreover, the integration of CsoS2A within the shell facilitates the assembly of larger shells (Fig. 3a through c), likely through stabilizing hexamer-hexamer association away from the curved shell vertices (Fig. 5e). This ultimately leads to the formation of larger α-CB shells than the CsoS2B-only shells.

Our study offers experimental evidence that highlights the potential strategies for the natural design of CsoS2 in native α-CBs for defining and regulating the shell architecture to ensure the structural plasticity of α-CBs. The evolution of six M-repeats in native CsoS2 may be of physiological significance to enable the α-CB shell to form a large and stable structure, for the recruitment and packaging of a large number of Rubisco via CsoS2-N, while maintaining a stable polyhedral shape by the least six motifs in CsoS2-M (Fig. 3c). Moreover, the multivalent interactions with shell proteins and the intrinsically disordered linker regions of CsoS2-M provide a means of modulating the assembly of shell proteins in a flexible mode and thereby modifying the α-CB shell structure (Fig. 5d). This regulating capability is further strengthened by the presence of two isoforms of CsoS2 (Fig. 5e). It is worth noting that while CsoS2 is crucial in regulating the size of the α-CB shell, it is not the only factor that determines the α-CB dimensions. The natural cargos enclosed within the CB and variations of shell proteins may also have a substantial impact on the overall size of naturally occurring CBs (45, 46). The structural plasticity of α-CBs, mediated by CsoS2 and other factors, may enable the host organisms to optimize their carbon fixation performance in response to varying environmental conditions.

In summary, this study provides mechanistic insights into the function of CsoS2, in particular, CsoS2-M, in shaping the formation and morphology of α-CB shells. Our findings advance the knowledge of the assembly principles of α-CBs and could inform rational design and reprogramming CBs and shell nanostructures for various biotechnological and biomedical applications.

## MATERIALS AND METHODS

### Generation of constructs

Primers (Table S1) for cloning genes and sequencing were ordered from Integrated DNA Technologies (US). All connections between genes and linearized vectors were achieved by Gibson assembly (Gibson assembly kit, New England BioLabs, UK). The pBAD vector was used as the backbone for expressing all shell operons. The shell-(*csoS2-NM*), shell-(*csoS2-NC*), shell-(*csoS2-C*), and shell-(∆*csoS2*) operons were generated by deleting nucleotide sequences encoding CsoS2-C, CsoS2-M, CsoS2-C and CsoS2, respectively, from the synthetic shell operon derived from *Halothiobacillus neapolitanus* (20). For the construction of variant shell operons expressing CsoS2 shells with varying numbers of M-repeats, the sixth repeat (M6) containing RFS was constantly retained as the last M-repeat in the CsoS2-M to ensure the production of two isoforms. By contrast, for operons expressing CsoS2B shells, M6 was exclusively removed.

The shell-(*csoS2B-only*) operon was generated by replacing the *csoS2* gene, in the shell operon, with the *csoS2B* gene (42), respectively. To construct fluorescence-tagged shell operons, the gene encoding mCherry was fused to the C-terminus of the *csoS1B* gene in various shell operons. The enhanced *gfp* gene, with the nucleotides encoding CsoS2-M, or CsoS2A fused at the C-terminus, was cloned into pCDFDueT-1 linearized by NcoI and XhoI. These constructs were placed under the control of a pTrc promoter, resulting in the generation of the pCDF-GFP-CsoS2M and pCDF-GFP-CsoS2A vectors, respectively. All these constructs were verified by PCR and DNA sequencing and transformed into *E. coli* Top10 and BW25113 cells.

### Expression and isolation of α-CB shells

*E. coli* BW25113 strains containing various *cso* vectors were cultivated at 37°C in lysogeny broth (LB) medium containing 100 µg mL^−1^ ampicillin. The expression of these vectors was induced by L-arabinose (1 mM, final concentration) once the cells reached an early log phase, corresponding to anoptical density at 600 nm (OD_600_) of 0.6. Cells were grown at 25°C for 16 hours with constant shaking and then were harvested by centrifugation at 5,000 *g* for 6 minutes. The cell pellets were washed with TEMB buffer (10 mM Tris-HCl, pH = 8.0, 1 mM EDTA, 10 mM MgCl_2_, 20 mM NaHCO_3_) and resuspended in TEMB buffer supplemented with 10% (vol/vol) CelLytic B cell lysis reagent (Sigma-Aldrich) and 1% protein inhibitor cocktail (100×) (Sigma-Aldrich).

The CsoS2 shells and CsoS2B-only shells were purified following the standard shell purification procedures at 4°C. The cell suspensions were lysed by sonication, and cell debris was removed by centrifugation at 12,000 *g* for 10 minutes, followed by centrifugation at 50,000 *g* for 30 minutes to enrich shells. The pellets were resuspended in TEMB buffer and then loaded onto sucrose gradients (10%–50%, wt/vol) followed by ultracentrifugation (Beckman, XL100K ultracentrifuge) at 105,000 *g* for 30 minutes. The CsoS2-C shells and ∆*csoS2* shells were purified following the mini-shell purification protocol described previously (32).

To isolate shells with varying numbers of M-repeats, we slightly modified the purification procedures from the standard protocol. These adjustments were made to ensure that the shells were distributed within the 10%–50% sucrose fractions. For shells with no more than five M-repeats, the purification process involved removing the cell debris, then subjecting the supernatants to centrifugation at 50,000 *g* for 30 minutes, followed by ultracentrifugation at 105,000 *g* for 1 hour. Shells with seven M-repeats were purified using the standard purification protocol. For shells with 10 M-repeats, after removing the cell debris, the supernatants were subjected to centrifugation at 50,000 *g* for 30 minutes, followed by ultracentrifugation at 105,000 *g* for 15 minutes. Each sucrose fractions were collected and stored at 4°C.

### Expression and isolation of GFP-loaded α-CB shells

*E. coli* BW25113 strains co-expressing shells and GFP-tagged proteins were cultivated at 37°C in LB medium containing 100 µg mL^−1^ ampicillin and 50 µg mL^−1^ spectinomycin. The expression of GFP-fused cargos was induced by the addition of 0.1 mM isopropyl-β-D-thio-galactopyranoside (IPTG) at OD_600_ = 0.6. After 4 hours of induction of the expression of GFP fusions, the shell expression was induced by 1 mM L-arabinose, and cells were then grown at 25°C for 16 hours. The isolation of GFP-incorporated shells was purified following the standard purification protocol described above.

### SDS-PAGE analysis

SDS-PAGE was performed following the procedure described previously. Briefly, 40 µg of total protein was loaded into each well of 16% polyacrylamide gels and stained with Coomassie Brilliant Blue R250.

### Transmission electron microscopy

Thin-section transmission EM was performed to visualize the reconstituted shell structures in *E. coli* strains (47). Isolated shell structures were characterized using negative staining EM. Images were recorded using an FEI Tecnai G2 Spirit BioTWIN transmission electron microscope equipped with a Gatan Rio 16 camera. Image analysis was carried out by using ImageJ software. The shell diameter data were randomly collected from 100 shell particles on EM images. The diameter of each polyhedral shell particle was measured by drawing diagonals three times from various angles, all intersecting at the same center point, using ImageJ software, and the resulting measurements were then averaged. The irregularity degree was determined by calculating the ratio of the standard deviation to the average of three diagonal measurements for each shell.

### Confocal microscopy

Overnight-induced *E. coli* cells were immobilized by drying a droplet of cell suspension onto LB agar pads as described previously (48). Blocks of agar with the cells absorbed onto the surface were covered with a cover slip and placed under the microscope. Laser-scanning confocal fluorescence microscopy imaging was performed on a Zeiss Elyra 7 with Lattice SIM² microscope equipped with a 63×/1.4 NA oil immersion objective, excitation wavelength at 488 and/or 561 nm. GFP and mCherry fluorescence were detected at 500 nm–520 nm and 660 nm–700 nm, respectively. Live-cell images were recorded from at least three different cultures. All images were captured with all pixels being below saturation. Image analysis was carried out using ImageJ software.

### Statistics and reproducibility

All experiments reported here were performed at least three times independently and at least three biological repeats were performed for each experiment.

## SUPPLEMENTAL MATERIAL

Supplemental material mbio.01358-24-s0001.docx Supplemental figures and table.

ASM does not own the copyrights to Supplemental Material that may be linked to, or accessed through, an article. The authors have granted ASM a non-exclusive, world-wide license to publish the Supplemental Material files. Please contact the corresponding author directly for reuse.
